# A farewell message of the *editor-in-chief*

**DOI:** 10.1080/01652176.2023.2231520

**Published:** 2023-07-11

**Authors:** Han van der Kolk

**Affiliations:** ISME, University of Bern, Bern, Switzerland

Dear reader,

*The Veterinary Quarterly* is an international open access journal, which publishes review articles and original research in the field of veterinary science and animal diseases.

**Figure 1.**
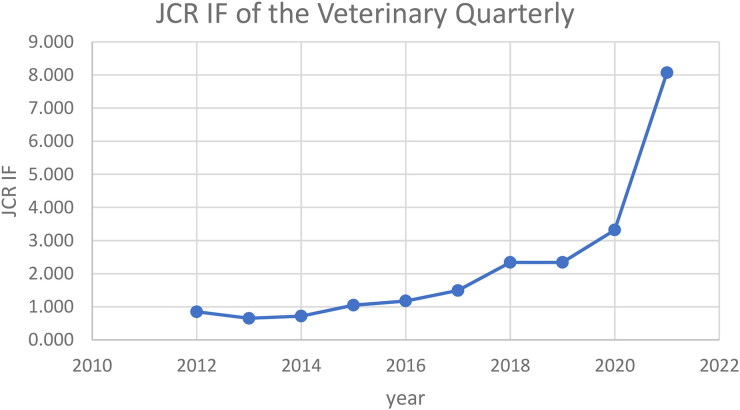
JCR impact factor (IF) of *the Veterinary Quarterly* over the years following its relaunch in 2011.

Since January 2011, following the relaunch of *the Veterinary Quarterly*, I served *the Veterinary Quarterly* as *editor-in-chief*. Over those twelve years the JCR Impact Factor increased from 0.850 to 8.071 (2021 Journal Impact Factor; Figure 1) ranking the journal in the top of the Veterinary Sciences JCR Category [3/144] to date. Of course, this steady increase in *the Veterinary Quarterly* JCR Impact Factor is largely attributed to the contributions and loyalty of our authors, reviewers and editorial board members.

As I shall resign as *editor-in-chief* of *the Veterinary Quarterly* on 30 June 2023 this is a good opportunity to sincerely thank all authors, reviewers, and editorial board members of *the Veterinary Quarterly* as well as the administration and editing teams at Taylor and Francis, the journal’s publisher for providing excellent support.

I would like to name two persons especially, namely Kuldeep Dhama and Merran Govendir.

Dr Dhama started to publish for *the Veterinary Quarterly* in 2014 and till to date he has published 44 papers in total for the journal. This effort by Dr Dhama and his excellent team is greatly acknowledged and certainly contributed substantially to the increase in the JCR Impact Factor of *the Veterinary Quarterly* and illustrates his passion to serve science.

Prof Govendir stepped in as an associate editor for small animal medicine also in 2014. Her thorough and timely processing of submitted manuscripts also contributed substantially to the increase in the JCR Impact Factor of *the Veterinary Quarterly*.

Besides, it was stimulating and a great pleasure working with you both.

The journal now faces new challenges such as, to my opinion, maintaining its position in the top of the Veterinary Sciences JCR Category combined with enhancing manuscript processing speed.

The opportunity to serve *the Veterinary Quarterly* as *editor-in-chief* has been an honour and privilege.

I wish my successor every success!

Sincerely Yours,


Han van der Kolk *ISME, University of Bern, Bern, Switzerland* j.h.van.der.kolk@umcg.nl



